# Obstructive Sleep Apnea and P300 Evoked Auditory Potential

**DOI:** 10.1590/S1808-86942011000600004

**Published:** 2015-10-19

**Authors:** Carlos Henrique Martins, Ney de Castro, Orozimbo Alves Costa Filho, Osmar Mesquita de Souza Neto

**Affiliations:** 1 PhD in Medicine – Graduate Program – Medical School of the Sta. Casa de S. Paulo; Assistant Physician at the Department of Speech and Hearing Therapy of the Dentistry School of Bauru - UNESP; 2 PhD in Medicine; Adjunct Professor; 3 Full Professor at UNESP – Bauru Campus; Director at the Department of Speech and Hearing Therapy at the Dentistry School of Bauru - UNESP; 4 PhD in Medicine – Graduate Program – Medical School of the Sta.Casa de S. Paulo; Adjunct Professor of Otorhinolaryngology of the Sta.Casa de S. Paulo

**Keywords:** auditory, cognition, event related potentials, evoked potentials, p300, obstructive, respiration disorders, sleep apnea

## Abstract

**Abstract:**

The obstructive sleep apnea syndrome (OSAS) reduces attention span, memory and concentration capacities, all associated with cognition. The analysis of the auditory P300 parameters could help infer cognitive dysfunction.

**Objective:**

To compare the data from polysomnography and the auditory P300 in adults, primary snorers with OSAS patients.

**Materials and Methods:**

Prospective study with primary snorers (N=12) and in OSAS patients (N=54), submitted to polysomnography, defined by the apnea-hypopnea index (AHI). The polysomnography and P300 variables were compared by the t-Student test, the Exact Fisher's Test, logistic regression and analysis of correlation with a significance level of 5%.

**Results:**

AIH had an inverse correlation with the oximetry in both groups. The P300 prevalence was lower in the OSAS group (Fisher's Exact Test, *p*=0.027). Patient age did not influence the P300 prevalence (regression analysis; *p*=0.232). The P300 amplitude was lower in the OSAS group (t-Student test; *p*=0.003) and the P300 latency was similar in both groups (t-Student test; *p*=0.89).

**Conclusion:**

The reduction in the P300 amplitude in patients with OSAS suggests cognitive dysfunction induced by a reduction in auditory memory.

## INTRODUCTION

The Obstructive Sleep Apnea Syndrome (OSAS) – causes a change to normal sleep architecture, with sleep fragmentation, hypoxia and frequent awakenings. Chronic sleep deprivation and fragmentation reduce its restorative capacity, induce excessive daily sleepiness, and reduce attention span, reduce memory and concentration, amongst other symptoms. Attention span, memory and concentration capacity are closely related to cognition^1^.

On the other hand, the auditory *P*300 is an electrophysiological event, depending on a previous acoustic experience, and it is a sensorial perception brain process. It is triggered by the individual's reaction to a previously established acoustic stimulus, it reflects the conscious interaction of the auditory system with the somatosensorial cortical area and requires the active mental participation of the patient. By analyzing its parameters, the *P*300 - known as a neuropsychobiological event – one can infer about the cognitive function in an objective fashion^2^,^3^.

Case-control clinical studies carried out with individuals subjected to a forced sleep deprivation showed latency extension and reduction in the auditory *P*300 amplitude in the study group. These results have suggested that the *P*300 changes in the study group were reflexes from lowering the state of awareness, which extends the reaction time^4^, ^5^, ^6^. The cognitive deterioration induced by sleep deprivation is a consequence of neurophysiological changes, demonstrated by reduction in amplitude and increase in *P*300 latency.

OSAS repercussion studies about *P*300 parameters are rare. Primary snorers and people with mild OSAS do not have changes to *P*300 latency and amplitude; patients with moderate to severe OSAS have an increase in *P*300 latency^7^, ^8^, ^9^, ^10^.

## OBJECTIVE

To assess changes in *P*300 amplitude and latency among primary snorers and patients with OSAS classified by polysomnograms.

## MATERIALS AND METHODS

This paper was approved by the Ethics in Research Committee of our Institution, under protocol number 117/2006. The individuals who participated in the study were given and agreed with the Informed Consent Form.

We assessed 66 patients, from both genders, in the age ranges between 22 and 59 years. The recording was prospective and sequential, between December of 2006 and November of 2007.

The individuals were divided into two groups; a control group (CG), made up of 12 snorers, and a study group (SG), made up of 12 primary snorers, and a study group (OSAS G.), made up of 54 people with OSAS. All 66 individuals were submitted to polysomnogram and were classified according to criteria from the American Academy of Sleep Disorders and from the Brazilian Association of Sleep Medicine^11^.

The following exclusion criteria were equally employed in both groups:

• Conductive hearing loss, mixed or sensorineural, unilateral or bilateral, with mean values at the eighths of the frequencies between 500 and 4000 Hz greater than 25 dBHL.
• External and middle ear disorder.
• Patients with neurological disorders or dementia^12^,^13^ and diabetes mellitus^14^.
• Inclusion criteria for the control group:
  — HAI: lower than 5 events/h
  — Average oxymetry higher than 88%
• Inclusion criteria for the OSAS group
  — HAI: greater than 5.1 events/h
  — OSAS diagnosis, except for other sleep disorders.

The polysomnogram was analyzed by the Meditron-Sonolab 620 device with 20 channels. The test was carried out at night, following the individual's circadian cycle, during physiological sleep. We recorded encephalography; electro-oculography; EKG; oronasal air flow; chest, abdominal and body position sensors; digital oxymetry; chin and anterior tibia electromyography.

The OSAS classification, according to the Hypopnea and Apnea Index (HAI), followed the criteria from the American Academy of Sleep Disorders and those from the Brazilian Association of Sleep Medicine^11^:

• Normal: HAI between 0 and 5 events per hour
• Mild OSAS: HAI between 5.1 and 15 events per hour
• Moderate OSAS: between 15.1 and 30 events per hour
• Severe OSAS: greater than 30.1 events per hour. The oxyhemoglobin saturation was considered

normal when it was higher than 90%; should it be lower than this, without exceeding the 3% of the Total Sleep Time (TST).

In order to assess the auditory *P*300, we used the device from Biologic's Evoked Potential System - version 6.1.0. The test was carried out between 8 and 10 o'clock in the morning, in a silent room, with the use of 3A “in the ear” phones. We utilized disposable electrodes; the active electrodes were placed on Cz and Fz; the reference electrode was on A_2_; and the ground electrode was placed on Fpz, according to international references^10^, ^11^, ^12^, ^13^, ^14^, ^15^, ^16^, ^17^, ^18^, ^19^, ^20^; we used impedance equal to or lower than 2 kΩ between the electrodes. The test technique was the target stimulus model. The target stimulus was the *tone burst* in the frequency of 2 kHz, randomly presented, in the probability of 20% of the stimuli; the frequent stimulus was made up by the *tone burst* in the frequency of 1kHz, with 80% probability of presentation. The series of stimuli was of 250 stimuli at an interstimuli frequency of 1/s. The sound intensity of both stimuli was of 70 dB SPL and binaural. The response triggered by the perception of the stimuli was motor, requiring individuals to raise one of the fingers in their hands when they perceived rare stimuli.

In assessing the results, we considered the Fz register as standard for reproducibility and the Cz register for wave analysis.

The *P*300 was measured in amplitude and latency. The amplitude in microvolt (μv) was defined as being the potential difference between the baseline and the apex of the positive wave. Latency in milliseconds (ms) was defined as the period of time between the onset of the stimulus all the way to the wave apex.

In the presence of *P*300, we created the latency and amplitude tables, according to age, the HAI and oxyhemoglobin saturation in building scatter graphics.

The study of the *P*300 prevalence between the two groups was carried out by the Fisher's exact test. The *P*300 prevalence and age range analyses were carried out by the simple regression logistics test.

We carried out *t-Student* tests for independent samples, in order to compare the values of *P*300 latency and amplitudes, between the two groups.

In the applied statistical tests, we used a 5% significant level (α = 0.05).

## RESULTS

The CG had six men and six women; the mean age was 34.41 years, ranging between 22 and 54 years.

The OSAS Group had 46 men and eight women; with a mean age of 43.53 years, ranging between 24 and 59 years.

In the control group, the mean HAI was 2.61/h and it happened between 1.2/h and 4.6/h. The mean nadir of oxyhemoglobin saturation was 88%, and it varied between 77% and 94%; saturation below 90% did not exceed 2.7% of the TST.

In the OSAS group, the mean HAI was 39.51/h and it varied between 5.4/h to 106.7/h. The mean oxyhemoglobin saturation nadir was 79.07% and it varied between 61% and 98%; saturation below 90% had a mean percentage value of 18.64% of the TST.

**Figure 1:**
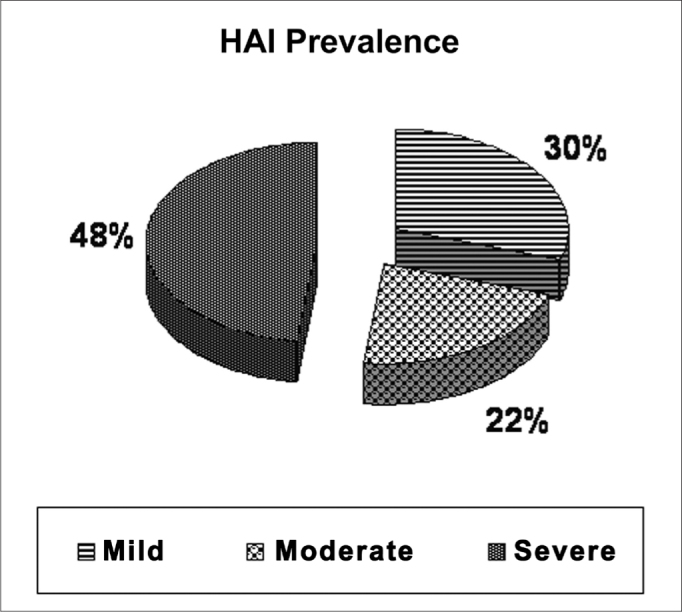
Distribution of the OSAS severity degree, according to the HAI.

The percentage distribution according to the apnea severity in the OSAS group was mild in 30% (16/54), moderate in 22% (12/54) and severe in 48% (26/54) (Figure 1).

*P*300 was recorded from all individuals in the CG. The mean latency was 303.56ms and it fluctuated between 242.80ms and 347.42ms; the mean amplitude was 10.40μV and it oscillated between 5.88μV and 18.7μV.

*P*300 was recorded in 66.67% (36/54) of the individuals from the OSAS group. Mean latency was 329.29ms; and it varied between 227.18ms and 463.49ms; the mean amplitude was 6.77μV and it varied between 2.22μV and 14.60μV (Table 1).

**Table 1:** P300 prevalence in the CG and OSAS group.

	P300 Prevalence	
	present	absent
C.G	12	0
OSAS G.	36	18
Total	48	18

Fisher's Exact Test: *p*=0.027 (significant).

**Figure 2:**
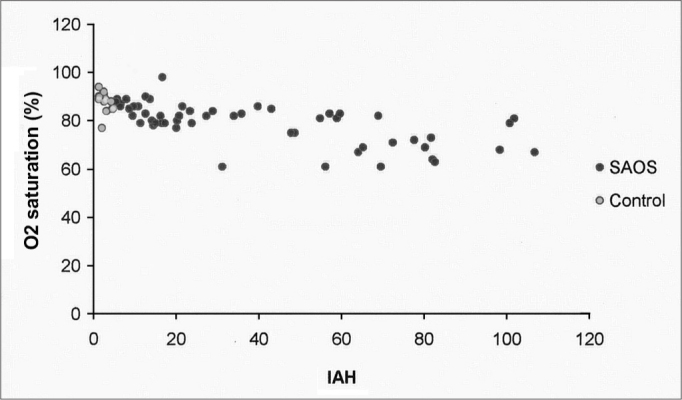
Oxymetry mean saturation distribution, according to the HAI in both study groups.

In the scatter charts of *P*300 variables (amplitude and latency) and considering the polysomnogram (HAI, oxymetry), there was an inverse correlation between HAI and the oxymetry (Figure 2).

The prevalence of the auditory *P*300 in the OSAS group (66.7%) was lower than that in the CG (100%) (Fisher's Exact Test; *p*=0.027).

In both groups, age did not influence the *P*300, through the logistic regression analysis (*p*=0.232) (Table 2).

**Table 2:** P300 prevalence, according to age range in the CG and OSAS group.

*P*300 Prevalence				
	Control Group and OSAS Group			
Age range	Present	Absent	Present	Absent
20 -30	5	0	4	1
30 -40	5	0	6	2
40 -50	0	0	16	8
50 -60	2	0	10	7

Simple logistics regression: *p*=0.232.

Tables 3 and 4 show the *P*300 minimum and maximum values, the mean, median and standard deviation (SD) of age, oxymetry, and amplitude/latency in the CG and OSAS Group.

**Table 3:** Control group: mean, standard deviation; minimum, maximum and median values for age, oxymetry nadir and P300 latency/amplitude (N=12).

	Mean	sd	Minimum	Median	Maximum
Age	34.4	9.8	22.0	33.5	54.0
Oxymetry Nadir	88.0	4.4	77.0	89.0	94.0
Latency	303.6	28.1	242.8	307.6	347.4
Amplitude	10.4	4.2	5.9	9.7	18.7

**Table 4:** OSAS group: mean, standard deviation; minimum, maximum and median values for age, oxymetry nadir and P300 latency/amplitude (N=54).

	Mean	sd	Minimum	Median	Maximum
Age	43.9	8.9	24.0	45.5	59.0
Oxymetry nadir	79.1	8.3	61.0	81.0	98.0
Latency	329.3	48.5	227.2	324.8	463.5
Amplitude	6.8	3.1	2.2	6.4	14.6

The *P*300 amplitude was lower in the OSAS Group, by the mean amplitude difference between the CG and the OSAS group (mean difference =3.6uV; *p*=0.003; CI:1.3 – 5.8; t-student test); latency was similar in both groups, by the mean latency difference between the CG and the OSAS group (mean difference =25.7; *p*=0.089; CI: -55.6 - 4.1, t-Student test) (Table 5).

**Table 5:** Student-t test comparing Latency and Amplitude between the control and the OSAS groups.

Mean difference			Confidence interval (95%)	
C.G. – OSAS group		*p*	Lower limit	Upper limit
Latency	-25.7	0,089	-55.6	4.1
Amplitude	3.6	0,003*	1.3	5.8

p*: significant “*p*” value.

## DISCUSSION

Patients with neurologic disorders^12^,^13^ and diabetes mellitus^14^ were taken off the study because of *P*300 changes caused by these disorders.

The hearing loss exclusion criteria was established in order to avoid biases; individuals with peripheral hearing loss may influence the *P*300 and compromise the obtained results^15^.

The *P*300 was executed following the individual's circadian cycle in the morning, when the awareness and concentration status are ideal and favor the *P*300 generation^16^,^17^. The response required from the target stimulus was motor, in order to maintain the state of awareness - factors which favor the *P*300 acquisition^12^,^18^.

In the present study, the CG, made up of primary snorers, had a prevalence of 58.33% of individuals with more than 40 years, and in a 1:1 ratio, as far as gender is concerned. The OSAS Group had a prevalence of 90.75% of individuals with more than 40 years, and in a 5:1 ratio favoring males. This age and gender prevalence in the OSAS group has been reported by most authors^1^,^19^,^20^. The decision to use primary snorers in the primary snorers CG, instead of asymptomatic volunteers, brought about an unexpected bias as far as age is concerned: the mean age of the CG (34.41 years) was lower than that of the OSAS group (43.52 years). The likelihood of a snorer with more than 40 years of age having OSAS is higher and proportional to age^1^,^13^, and this made it difficult to have a CG sample of equal age range from that of the OSAS group.

The analysis of the scatter chart between the *P*300 and polysomnogram variables in both groups showed that the only variables which had a correlation were HAI and oxymetry; such correlation was inverse, and the higher the HAI, the lower the oxyhemoglobin saturation values. The brain cortex and the hippocampus, *P*300 generation structures, were described as being particularly sensitive to hypoxemia^21^; and hypoxemia may induce neuronal lesions and hippocampus atrophy, which restricts the neurocognitive performance^22^.

The *P*300 prevalence was significantly lower in the OSAS Group – 66.67% (36/54). Such data may be justified by the fact that 70% of the individuals with OSAS had the moderate and severe types; therefore, with a greater impairment in awareness, attention and concentration^3^,^23^. On the other hand, there are papers reporting *P*300 changes in individuals with OSAS; however, without mentioning the absence of such potential^7^, ^24^.

The *P*300 amplitude in the OSAS Group was significantly lower. The amplitude reflects brain activity in the parietal-temporal and pre-frontal areas, associated with the auditory memory^6^,^15^ which would be reduced in individuals with OSAS.

In the present study, the *P*300 latency was not a sensitive parameter in OSAS patients. Latency is associated to the interstimuli frequency to the individual's attention and concentration^15^,^25^. The studies which showed a significant delay in *P*300 latency had individuals with severe OSAS^13^, of sleep deprivation associated with forced awakeness^6^ and because of their circadian cycle phase^17^.

On the other hand, a study involving individuals with OSAS showed that the attention deficit is more severe in young people, when compared to elderly (threshold age: 50 years), and that age it does not interact with OSAS in worsening the cognitive deficit^26^.

## CONCLUSION

The reduction in auditory *P*300 amplitude in the OSAS patients of the present study suggests a cognitive dysfunction, induced by the impairment in auditory memory.
